# Biomedical Ph.D. Students Enrolled in Two Elite Universities in the United Kingdom and the United States Report Adopting Multiple Learning Relationships

**DOI:** 10.1371/journal.pone.0103075

**Published:** 2014-07-23

**Authors:** Matthew W. Kemp, Benjamin M. Lazarus, Gabriel G. Perron, William P. Hanage, Elaine Chapman

**Affiliations:** 1 Graduate School of Education, The University of Western Australia, Perth, Australia; 2 School of Women’s and Infants’ Health, The University of Western Australia, Perth, Australia; 3 Ely, Cambridge, United Kingdom; 4 Department of Biology, University of Ottawa, Ottawa, Canada; 5 Department of Epidemiology, Harvard School of Public Health, Boston, Massachusetts, United States of America; University of Massachusetts, United States of America

## Abstract

**Objective:**

The ability to form multiple learning relationships is a key element of the doctoral learning environment in the biomedical sciences. Of these relationships, that between student and supervisor has long been viewed as key. There are, however, limited data to describe the student perspective on what makes this relationship valuable. In the present study, we discuss the findings of semi-structured interviews with biomedical Ph.D. students from the United Kingdom and the United States to: **i)** determine if the learning relationships identified in an Australian biomedical Ph.D. cohort are also important in a larger international student cohort; and **ii)** improve our understanding of student perceptions of value in their supervisory relationships.

**Study Design:**

32 students from two research intensive universities, one in the United Kingdom (n = 17), and one in the United States (n = 15) were recruited to participate in a semi-structured interview. Verbatim transcripts were transcribed, validated and analysed using a Miles and Huberman method for thematic analysis.

**Results:**

Students reported that relationships with other Ph.D. students, post-doctoral scientists and supervisors were all essential to their learning. Effective supervisory relationships were perceived as the primary source of high-level project guidance, intellectual support and confidence. Relationships with fellow students were viewed as essential for the provision of empathetic emotional support. Technical learning was facilitated, almost exclusively, by relationships with postdoctoral staff.

**Conclusions:**

These data make two important contributions to the scholarship of doctoral education in the biomedical sciences. Firstly, they provide further evidence for the importance of multiple learning relationships in the biomedical doctorate. Secondly, they clarify the form of a ‘valued’ supervisory relationship from a student perspective. We conclude that biomedical doctoral programs should be designed to contain a minimum level of formalised structure to promote the development of multiple learning relationships that are perceived as key to student learning.

## Introduction

The Ph.D. is the highest degree awarded prospectively by Universities, and has been described as the (p.*X*) ‘monarch of the academic community’ [1]. Setting aside professional doctorates, admittance to a Ph.D. program requires a demonstrated track record of excellence in undergraduate studies and a willingness to commit to a further, lengthy period (commonly between 3 and 7 years depending on country and institution) of intensive full-time study. In the past decade, a number of governments in the developed and developing world alike have instituted or continued programs that have seen substantial year-on-year increases (as much as 40% in some OECD countries between 1998 and 2008) in the numbers of students graduating from doctoral programs [2]–[4].

The significant expansion in the number of students undertaking doctoral studies has been accompanied by growing concerns over the lack of tenure-track positions for junior academics [5]–[7], high rates of student attrition [8], [9] and even the relevance of traditional doctoral programs to what many argue is an increasingly cross-disciplinary, output-focused knowledge market [10]. Despite its importance to society’s intellectual and economic well-being, doctoral education remains one of the least well understood areas of university higher education. As noted by Enders (p.125) ‘research training traditionally had – and to a considerable extent still has – an unclear status within the higher education system’ [11].

A number of investigators have undertaken studies that have demonstrated the importance of a range of student (racial identity, gender, socio-economic and cultural history, intelligence) and environmental (funding, course structure, supervision, learning relationships, motivation, clearly articulated study objectives, socialisation) factors to the doctoral learning environment. These important studies have served to highlight both the complexity of the doctoral learning environment and the need for greater understanding of how it functions on a student-specific level [9], [12]–[21].

The focus of the current report is on doctoral learning in the biomedical sciences. Strong evidence exists to suggest that cultures of academic practice (and thus learning) differ greatly across departments, institutions and disciplines [18]. Accordingly, the extrapolation of findings from one sphere of academia to another is often difficult. This is of particular concern for those with an interest in driving research-informed improvements to the scholarship of doctoral education in the biomedical sciences because, as noted by Leonard (p.4), ‘research on the doctorate has usually noted the disciplinary area(s), but tended to focus disproportionately on the social sciences and (especially) Education’ [22]. This sentiment is echoed by more recent work by Cumming, who notes that (p.877) ‘there is an acknowledged gap in the literature on doctoral education with regard the natural and physical sciences’ [23].

The relationship between students and supervisors has been the focus of a large number of research studies and is traditionally considered one of the most important elements to ensuring a successful doctoral project candidacy. In a study investigating factors impacting time to completion in doctoral degrees, Seagram, Gould and Pyke reported the critical need for students to meet (p.332) ‘regularly and frequently with their supervisors’ [24]. Girves and Wemmeraus listed the student/advisor relationship and financial support as being two factors fundamental to graduate education, noting that (p.165) ‘we believe that student relationships with faculty members are crucial to the student’s educational and professional development and ultimately to the student’s degree process’ [25]. Whilst acknowledging the substantial body of data supporting the importance of supervisory relationships, a number of investigators have argued for the need for a broader assessment of learning relationships in doctoral learning processes [26], [27]. Boud and Lee argue for investigators to take into account (p.514) ‘the actual material practices and relationships deployed by students’ [28]. Pearson and Brew argue for the importance of viewing research and research training as a social practice and cite earlier work by Delamont and colleagues who theorise the importance of student socialisation to research communities wherein learning takes place by student (p.142) ‘participation in the social practice of the community’ [29]. The argument for a greater understanding of how communities of practice support doctoral learning is echoed by earlier work by Lahenius, who concludes an excellent summary of the relevant literature by observing that (p.31) ‘there has been little theorisation on how communities of practice related to doctoral education’, stating that ‘greater understanding is still needed’ [14].

We argue that it is difficult to engineer meaningful improvements to doctoral learning in the biomedical sciences in the absence of a field-specific body of empirical data. This argument is supported by earlier commentary by Pearson who contends that (p.530), ‘without an accurate and nuanced understanding of the contemporary student population and the doctoral experience, its diversity and complexity, there is the danger of policies being put in place that do not advance the interests of doctoral students’ [30].

In an effort to contribute to the formation of a knowledge base specific to the biomedical doctorate, we have previously presented data suggesting that stable access to multiple learning relationships is a key factor in doctoral learning in the biomedical sciences [31], [32]. One important gap in the contemporary literature, identified in our previous work, is a lack of empirical data to describe the student perspective on what particular elements of their relationships with supervisors are valuable in supporting their learning. A number of investigators have focused on supervisors’ perceptions of what the process of doctoral supervision entails or how it is perceived [33]–[36]. However, despite the significant body of evidence available to highlight the importance of considering contextual, student-specific factors in doctoral learning processes, fewer studies have sought to clarify the valued aspects of supervision from a student perspective. This sentiment is reflected by the work of Lee and McKenzie, who suggest that (p.71) ‘there appears to be an absence of qualitative approaches that enable students to reflect and provide formative feedback to supervisors about the aspects of supervision that are important to them, within a well-informed framework’ [37].

In this vein, we have previously undertaken an analysis of learning relationships in the biomedical doctorate and concluded (p.383): ‘**i)** that relationships between Ph.D. students and non-supervisor peers are perceived as being at least as important as relationships between Ph.D. students and their supervisors; **ii)** that these relationships adopt qualitatively different functions within the biomedical doctorate; and **iii)** further study, specifically with regards the nature of student – supervisor relationships in the contemporary environment are urgently needed’ [31].

In the present study we discuss the findings of a series of semi-structured interviews with biomedical Ph.D. students in the United Kingdom (n = 17) and the United States (n = 15). In analysing these data we aimed to extend our earlier work in doctoral learning relationships to: **i)** determine if the learning relationships identified in an Australian biomedical Ph.D. cohort are also important in a larger international student cohort; and **ii)** improve our understanding of student perceptions of value in their supervisory relationships.

## Methodology

### Ethics Statement

Ethical approval was gained from the Humanities and Social Sciences Research Ethics Committee, University of Cambridge and the Harvard University Committee on the Use of Human Subjects, prior to participant recruitment commencing. Informed consent was obtained, in writing, from each participant prior to the interview commencing. Participants were advised of their right to withdraw from the study without prejudice. Participants were debriefed as to the study aims and objectives at the conclusion of their interview. All interviews were redacted and anonymized to ensure participant confidentiality.

### Characteristics of Doctoral Programs in the UK and US Cohorts

Diversity is recognised as one of the hallmarks of the Ph.D. student population [30], [38]. We argue that the same could be said for the structure of Ph.D. programs, with program length, taught module requirements, funding arrangements and examination criteria varying widely between institutions and countries. A detailed description of variant doctoral program structures is outside the scope of this work, and has been dealt with in depth elsewhere [1], [39]. Herein, we will focus on the structures of the doctoral programs represented in our study cohort.

### Characteristics of the UK Cohort

In the UK cohort, 14 students were female and three were male. Seven (six females and one male) students were international students from outside the UK. The average interview length was 33 minutes. The standard length of biomedical Ph.D. programs in the UK cohort was four years. The Medical Research Council (MRC) and the Wellcome Trust were identified as key funding providers. The MRC provides doctoral training grant funding directly to research organisations based on their MRC grant and fellowship income [40]. The Wellcome Trust funds Ph.D. Studentships at a number of top-ranked UK institutions under seven subject groupings (developmental biology and cell biology; genetics, statistics and epidemiology; immunology and infectious disease; molecular and cellular biology; neuroscience; physiological sciences; and structural biology and informatics) [41]. Students are recruited directly by individual, institutionally-based programs (31 were advertised in 2014) on an annual basis. In addition, a diverse range of philanthropic bodies including the British Heart Foundation and Cancer Research UK provide funding for disease-specific Ph.D. programs. Ph.D. enrolment applications for the institution in the UK cohort consisted of a combination of *curriculum vitae*, academic transcripts, an on-line application form, letters of reference and frequently a personal statement. Admission to Ph.D. programs (especially those funded by the Wellcome Trust) are advertised as being fiercely competitive, requiring a minimum of upper second class honours in a Bachelor’s or even a Master’s degree. Students from non-native English backgrounds were required to demonstrate proficiency in English.

As reflected in our UK cohort, Ph.D. programs frequently adopted a 1+3 year structure. In these programs, the first year is comprised of tailored induction courses focusing on ethics, laboratory safety, intellectual property, research processes and introductory lectures on program-specific theory. Students then rotate through two or three laboratory placements (generally 9–11 weeks in duration) offered by principal investigators involved in the doctoral program, during which they complete a mini-project and submit a report. After completing rotations, students then apply to join a laboratory group and prepare a detailed Ph.D. research proposal which is assessed, along with the rotation mini-reports, by internal and external examiners. Students also undergo an oral defence of their research proposal. Students who successfully complete the first year of their program then spend three years engaged in full-time research towards the production of a thesis. Thesis examination is by a panel of external examiners and the student also undertakes an oral defence of his or her thesis work. Students are expected to maintain a personalised progress log (including annual reports) which records scheduled meetings with supervisors in addition to the completion of a set number of compulsory academic and training modules.

### Characteristics of the US Cohort

In the US Cohort, 10 students were female and 5 students were male. One student was from outside of the US. The average interview length was 32 minutes. The length of doctoral programs varied significantly based on the program and the progress of the individual student. A number of programs from the US cohort institution reported a median time to completion of 5.5 years from enrolment. Several students in our US cohort were in their 6^th^ year of doctoral study. Programs were frequently interdisciplinary in nature and involved investigators based in a wide range of science departments and affiliated teaching hospitals.

Without directly identifying the doctoral programs described in this study, their characteristics may be summarised as follows. Admission is by competitive application to the co-ordinating program office. In general, applicants are required to submit an online application, *curriculum vitae*, academic transcripts, Graduate Record Examinations (GRE) general test results, a personal statement, three letters of recommendation and Test of English as a Foreign Language (TOEFL) results for non-native English speakers. Students judged by the program to be of an exceptional standard are subsequently invited for face-to-face interviews on campus. All students successful in gaining a place receive full tuition, health insurance and a stipend of up to $US 3,000 per month from their program.

On enrolment, students reported being assigned a program advisor to provide guidance with academic and non-academic support. A number of students also reported meeting infrequently with their program head. Their thesis research was directed by a primary investigator (PI; a senior, independently funded scientist responsible for the overall academic direction of student research) selected following laboratory rotations. Students also reported having a dissertation advisory committee comprised of three academics, tasked with the provision of guidance and feedback to the student and their PI.

Depending on the program, students spend approximately one year undertaking structured training (e.g. statistics, critical reading) and theory course work designed to support the development of subsequent thesis research. Simultaneously, students rotate between program laboratories (often two or three in total) in order to select a thesis topic, PI and a laboratory in which to work. In the second year, students are required to undertake a series of qualifying exams (often lasting several days) in order to advance to full Ph.D. candidacy. The qualifying examinations are designed to demonstrate proficiency in subjects studied in year one. Students are often required to complete a set number of hours working as an unpaid teaching assistant over the course of their studies. Students reported that final examination was by submission of a thesis and an oral defence of thesis research.

### Student Interviews

We employed a semi-structured interview model to investigate student perceptions of their doctoral learning environment. Semi-structured interviews are utilised extensively in the humanities and education disciplines [42], [43]. This approach allowed us to standardise our data collection methods and focus on specific areas of interest (learning relationships) whilst retaining the flexibility necessary to capture the diversity of responses and experiences that were likely to derive from doctoral students working in the biomedical sciences in two countries. The biomedical sciences were classified as disciplines in the life, natural or and health sciences. The common factor driving research in these areas was an aim to generate data necessary to improve human health and wellbeing.

We aimed to enrol 20 students from each institution in order to exceed the sample size required for response saturation, based on cohort numbers suggested in previous interview studies [44]. The 32 students in this study were drawn from two research intensive institutions, one each in the UK and USA. Participant recruitment was by departmental email lists or by personal referral. Interview questions were refined from those used in our earlier work in Australia [31], [32] to focus on the structure of learning relationships and perceptions of value in student – supervisor relationships.

The following questions were used to initiate students to reflect on their learning relationships:

- What are some of the important people or relationships in your life as a Ph.D. student? Why are they important?
- How do you go about learning the theory that underpins your work? Why?
- How do you go about learning the technique that underpins your work? Why?
- Describe your relationship with your supervisor.
- How much day-to-day input does your supervisor have in your work?
- Do you think this relationship is valuable for your time as a Ph.D. student? (why/why not? What makes it valuable?)

Interviews in the UK and US were undertaken in November 2012. One investigator (MWK) was present for all interviews in this study. 17 students from the United Kingdom and 15 students from the United States were recruited to participate in the study. Interviews were recorded in full and verbatim, de-identified transcripts were generated by one investigator (MWK). The fidelity of transcripts was determined by retrospectively comparing three, randomly selected 10 second excerpts from each transcript with the interview recording.

Transcripts were analysed for thematic responses using a Miles and Huberman notation and memoing approach as described by Punch [43]. Briefly, interview transcripts were initially analysed by a single investigator (MWK) to identify both isolated and repetitive themes within individual transcripts. Once all transcripts were analysed, the presence of inductively identified themes was then assessed across all interview transcripts. A categorical list of these themes was constructed, based upon the frequency of thematic identification across the data set. Interview transcripts were then re-analysed to investigate the perceived structure and importance of each individual theme to the construction of each student’s learning environment, with specific focus on learning relationships as appropriate for the objectives of the present study. Care was taken to report both major and minor themes emerging from the data set. In undertaking this analysis we applied a number of techniques suggested by Wright, Murray and Geale for undertaking rigorous qualitative data analysis, including; adopting a balanced approach to data interpretation, transcribing all interviews in verbatim, providing contextually rich quotations as evidence of data, and maintaining an attitude of scepticism by not coming to a final conclusion regarding the importance of identified themes until all data had been thoroughly analysed [45].

## Results

In keeping with the results of or our earlier studies in an Australian Ph.D. cohort, students in the UK and US cohorts reported that interactions with postdoctoral (post docs), technical staff, other Ph.D. students and supervisors constituted their main learning relationships. The data that support the existence of three key relationships in the biomedical doctorate are presented, along with a brief summary, in the Results section. These data are then discussed, with reference to the existing literature, in the Discussion and Conclusions sections that follow.

### Technical learning is predominantly facilitated by relationships with postdoctoral researchers, other students and technical staff

A majority of students (26 out of 32) reported that other relationships with postdocs or more senior Ph.D. students were the primary means of obtaining the technical knowledge they required to execute their experiments. As described in greater detail in the discussion section, these data reinforce earlier studies suggesting the importance of relationships between doctoral students and non-supervisory peers in facilitating technical learning in the biomedical doctorate:

**UK04:** *Mainly through lab technicians or senior members of the lab, post docs.*

**UK05:** *During the Ph.D. we learn from the post docs so they pass on their knowledge of the specific techniques for the Ph.D.*

**UK09:** *And I think the other important relationships are I guess lab members, you know, the post docs and the Ph.D. students in the lab. Apart from that, no I think those are the main ones. In terms of every-day things like setting up equipments, or of thinking of new experiments to do, to run ideas by and, you know, trouble shoot things, find out what is going wrong, that usually tends to happen, just discuss the science.*

**USA08:** *Either I’ve had to develop the techniques myself or go to, usually the post docs, in my lab.*

**USA11:** *Afterwards there is the other postdocs. And until recently I was the only graduate student of my lab, my lab is a big lab so it is very hard to make your place and so the other postdoc were very important because they were the only people I could deal with and also because they were the one that could give me the training.*

**USA15:** *So most of the time if I need to learn a technique I’ll find a postdoc that knows how to do it and, like, beg them for help.*

Similarly, 24 of 32 students reported that their supervisor(s) had little or no involvement in teaching them the laboratory techniques required to undertake their studies.

**UK02:** *He’s very hands off in terms of practical work, he’s really hands on in terms of philosophy and what I’m doing. So he knows what I am doing, but he is not going to come to the lab to see what I am doing.*

**UK08:** *So if there is something like I need a signature or a quick question I can always pop in and ask him, but he is not involved in the day to day research.*

**UK09:** *So in terms of the method I think with PIs since they have been doing the theoretical or like the thinking side of things so much they tend to forget how things work in the lab and how long things take so it’s, I guess you always have to keep reminding them or get help with the methods from someone else in the lab rather than the PI.*

**USA01:** *He’s not in the lab at all. He, um, he’s actually started coming back to the lab which is quite unusual for him. It is very admirable on the other hand. And he doesn’t actually have a lot of experience with experimental techniques because he’s an M.D., He’s only finished his Ph.D. for two years. I probably have more practical hands on experience than he does. So, yeah, in terms of lab, not really, It’s only weekly meetings where we discuss the experiments.*

A small number (3 of 32) of students reported that their supervisors played an important role in them learning laboratory technique. In this scenario, the supervisor was usually a junior member of academic staff and was responsible for a small laboratory group.

**UK03:** *My supervisor, […] has shown me pretty much everything she’s been really good. And then when she was on maternity leave I got one technique was shown to me by my post doc, […]. But personally I prefer […] to teach me because she is a lot more thorough.*

**USA14:** *We’re in a, I’m in a small and new lab. And my advisor is pretty hands on so I actually work directly with him. Whereas other people I have talked to in other types of labs have had a more distant experiences. I was just told a story the other day about someone in a lab of a very famous person who met with their advisor I think twice during their entire Ph.D. project, whereas I meet with my PI probably on average four or five times a week.*

When students did report difficulty in accessing the technical learning relationships they viewed this as having a negative impact on their doctoral progression.

**US12:** *We don’t have that much expertise. So we tend to rely on postdocs from the […] lab or from the […] lab or from other labs around there. There’s been recently more, a couple of new post docs that know a bit more of yeast-specific experimental techniques. So that has been useful. But I still think we are we are a little bit lacking on that department, in the lab. It makes my work more difficult. It is also a little awkward to ask for help in other labs, sometimes. In the […] lab, they were really nice in the beginning, but you can see sometimes they get irritated when you come and ask because you are not in their lab and it’s. […] has, […] is my PI, he has a weird, not very defined relationship with […], in which their lab kind of supports us but not really, and it’s very a kind of grey area, to judge exactly when to ask for help and when it is not OK. So and the lab manager gets pissed off at you. It’s very weird.*

### Empathetic emotional support is predominantly facilitated by relationships with other students

In our cohort, the unique contribution of inter-student relationships to doctoral learning was the provision of emotional support and a sense of camaraderie. Although support and positive feedback was an important component of the student-supervisor relationship (see below), the nature of this emotional support was qualitatively different to that provided by inter-student relationships. Students reported the importance of being able to talk to students about their shared experiences. The unique feature of this support was that other Ph.D. students were viewed as able to empathise and reflect upon each other’s situations, providing feedback, perspective and reinforcement coloured by similar levels of learning experience.

**UK03:** *And it is really nice having […], the other Ph.D. student of […] and […]’s ‘cos it’s really good, like, having her for moral support I suppose when they’re both putting too much work on us at least we can both feel like oh god, have you done this?*

**UK08:** *Then, like, I share an office with five other people, most of them are Ph.D. students as well and those relationships are very very helpful to me. Probably maybe more than my supervisor, actually. I mean my supervisor is great, not criticising him. But just because on a day to day basis then they, ah, I dunno, we just kind of support each other as we’re all going through the same thing and in particular, so, she’s just left actually, but a Ph.D. student in the year above me, a lot of concerns and difficulties that I was coming across and she could totally understand those because she had been through them before and was able to advise me and kind of show me that I wasn’t alone in the worries and things I was having.*

**UK 11:** *Then I have friends in the department who are other students, and that sort of stuff. So that’s a kind of good support resource just to talk about how things are going and commiserate when things are going badly.*

**USA04:** *I do have a couple of friends that, you know, who are kind of going through the same things, so when I am like, oh god, I feel like an idiot, literally every day and I don’t know what I am doing then they are like oh me too and I am like oh good I’m not the only one who is totally clueless. It is, ah, a relief to be in the same boat with other people and just relate, from just the relatability there.*

**USA06:** *So luckily we have a very large programme here in my program the one that I am in, […], and so our class is something like fifty students. And so that provides a very large and rich community, and I’m fortunate to have some very good friends and we all support each other and look out for each other and talk to each other about science, about outside of science things and get together.*

### Coordination, confidence, and cash – what students perceived as valuable in a supervisory relationship

Most students (30 out of 32) described their relationship with supervisors as being either very important or the most important to their work as doctoral students. In addition to a small number of students who reported gaining technical expertise from their supervisors (above), relationships were perceived as valuable because they provided the student with access to high-order project guidance, networking and finance and/or emotional support. These findings are in keeping with our previous work with an Australian cohort of Ph.D. students wherein we identified students forming guidance focused, technically focused and emotionally focused relationships with their supervisors [31].

**UK01:** *I think you need a good relationship with your supervisor. So that is definitely key.*

**UK02:** *Definitely my supervisor, obviously he is the person who decides everything I’ll have to do.*

**UK03:** *I think my supervisor is like, number one, the most important.*

**USA03:** *So my professor, […], she’s obviously pretty important ‘cos, we meet once a week, and then we go over what I’m doing, and what I am going to do and just go over all big picture things.*

**USA04:** *I think probably the first and foremost obviously would be my advisor, who runs the lab.*

**USA07:** *Who’s important to my Ph.D. career? So I think that there is a huge number of people that are important, it’s um, this is going to be a long list. Um, but my advisor is essential.*

Rather than technical support, the higher-order guidance provided to students through their relationships with supervisors consisted primarily of intermittent meetings to review experimental progress and discuss potential future avenues of investigation.

**UK17:** *My guy here is not that much help, to be frank, practically, but very good more in terms of this is where you should go. This is the direction you should go. This direction is not working out for you. You should change topics. But in terms of what experiments he is not much help. So I find that kind of experience to be the most valuable, not that they are smarter, not that they are have more degrees or anything like that or more papers, it is that they have more experience to guide you through your life.*

**USA04:** *So the day to day stuff is not something that I would tend to bother him with that is usually what I will go to postdocs for.*

**USA13:** *We will have guidance but he is not going to tell us what to do and so we need to take our projects into our own hands.*

The majority of students met with their supervisors on a weekly, fortnightly or even monthly basis. Students often reported that the frequency of their meetings reduced over the development of their projects.

**UK02:** *We try to have meetings every week. He is very good at this. If I’m lazy and don’t go to see him he remembers that I didn’t go, he is pretty good at this.*

**UK14:** *With my supervisor I would say towards the start of my Ph.D. it very much started off as weekly meetings, every single Monday, updating on the sort of progress, saying this is what I have done in the past week and him saying in the following week you might want try and get this completed and going over any particular areas that there might be a problem. I’m now currently starting my third year and gradually as you go on that becomes slightly more and more autonomous, so the frequency of the meetings goes down.*

**USA10:** *She’s fairly hands off in the lab, um, but, she, we meet with her, everyone in the lab generally meets with her on a weekly basis.*

**USA13:** *So he sets aside an hour a week at least for each of us and if we need more time we can go over et cetera but we meet once a week and ideally you have something to talk about.*

A smaller number of students, notably those who were supervised by senior academic staff, reported periods of several months between meetings.

**UK07:** *The other guys is at […]. So he is much more senior. And at the moment he is in New York on Sabbatical so I occasionally Skype him. So he’s, ah well, speak to me in four months’ time. And we’ll see what’s going on. So in terms of him that’s how he works.*

Conversely, the small number of students supervised by junior academic staff (usually responsible for small laboratory groups) reported that their relationships were patterned by a series of often-daily interactions as opposed to formalised meetings.

**UK03:** *With […], we’re in contact all the time. ‘Cos she’ll help me out with the actual experiments in the animal house always. We have meetings, there are no set times, we try for them to be, you know, say I have just got a batch of work done we will make sure we have meeting before I get to the end that we can figure out where I am next going.*

Access to research networks was viewed by students as being a highly valuable element of their relationship with their supervisor. Students viewed that this networking ability allowed access to research grant funding, specialist equipment and improved their chances of obtaining employment following completion of their studies.

**UK04:** *I guess the funding is very important. Biomedical research it is very expensive. What I do every day, everything costs money, and it’s his money and it’s his research in a way, I’m just sharing and taking part in it.*

**USA01:** *He’s determining, you know he gets the funding for the work that I do and it is his lab.*

**UK07:** *So because they are really plugged into this network already that means that I can get access to loads of stuff in […]. If you had a brand new supervisor who’s just new to […] then you wouldn’t have any of these connections. So you’d be just stuck in a bit of an isolated position. That’s the problem with […] actually, I don’t know if people have mentioned this, but there are many facilities here but it’s quite closed so you have to know the right people.*

**UK05:** *And also I think not so immediately but later on it would be I would say the benefit of her knowledge of the field and the people to help me find the lab for the post doc, so it’s one of her specialties that she has a very good network and good contacts, good connections in the field with the other experts. So I think that’s a big benefit.*

**USA12:** *But as, he might be more useful in the future when I need connections for recommendation letters.*

Emotional support was the final critical element of the supervisory relationship identified by students in our cohort. As noted earlier, the nature of the emotional support gained via supervisory relationships was, in almost all cases, qualitatively different from that deriving from relationships with other students. One student reported obtaining emotional support similar to that obtained with their relationships with other students.

**UK03:** *She’s approachable, I’ve cried on her, and she said that if it is ever too much and I am putting too much work on you, you can say. She’s like a friend. I think I’m really lucky. I don’t think most people have that.*

In contrast, a much larger number of students reported that the emotional support derived from their relationships with supervisors was in the form of a sense of structure, intellectual security and confidence that derived from working with or under a more senior academic with a successful track record in research.

**UK01:** *Before a Ph.D. at university you get very regular feedback as to how you are doing and where you are with the rest of the year group, you get exam results and that’s fine, but when you start doing your Ph.D. all that goes away and you have this massive chunk of time and you can do with it what you please and sometimes there are checkpoints and other times not so much. So what is useful is meeting the supervisor is that you have short term goals that are set and then you are able to say have a tick list which is important when you are away doing something for four years.*

**UK07** ***:*** *So he’s kind of overseeing it and the advantage is that he’s been in it for over forty years so if he was really concerned he’d tell me straight away. So clearly he is not concerned too much.*

**USA04:** *He has been around a while and has very good advice in terms of his own past and being in the field for a long time and so he can offer good advice to me as someone who doesn’t really know what I am doing ever.*

**USA06:** *His willingness to support my floundering efforts. And he doesn’t castigate me when things fail, at least hasn’t yet, he’s more like hey well you are not trained, you have to learn, it is OK, don’t worry about it so much. So that is also more like an emotional support kind of function as opposed to just an academic support kind of function.*

**USA07:** *It’s important because I am a young scientist learning how to be a scientist, and […] is teaching me in an excellent way.*

**USA08:** *Well I think motivation and positive feedback is pretty important, whether things work or not, just having someone else who is excited about the project besides me, is really helpful.*

**USA14:** *Yes a sense of security and a sense that, well it is reassuring to have someone else tell you that what you have said makes sense, as opposed to trusting only your own intuition, and as a student fairly early in my Ph.D. I don’t have all that much self confidence that what I have thought up on my own is valid.*

## Discussion

In undertaking the present study we had two aims: **i)** to determine if the learning relationships identified in an Australian biomedical Ph.D. cohort were also perceived as being important in a larger international student cohort; and **ii)** to better characterise students’ perceptions of value in their supervisory relationships. We suggest that the data presented in this study provide further evidence for the importance of multiple learning relationships in the biomedical doctorate. Additionally, these data serve to clarify the unique and important contribution that students’ relationships with peers (other Ph.D. students), postdocs, technical staff and supervisors each make to doctoral learning processes in the biomedical sciences.

In contrast to the traditional student – supervisor model, a number of researchers have framed doctoral learning within the context of a community of practice [14], [23], [28]. In a study investigating Ph.D. student perceptions of small group learning, Lahenius (p.30) cites Lave and Wenger to define communities of practice as ‘a system of relationships between people, activities and the world; developing over time, and in relation to other tangential and overlapping communities of practice’ [14], [46]. We contend that the data in our study suggest the existence of a similar learning community in the biomedical sciences, an assertion in keeping with Cumming’s view that (p.888) ‘Not only is a candidate’s learning and research influenced by individuals beyond the research group, but supported by human and physical resources from a much broader base as well’ [23].

As in our original Australian cohort, interactions with peers (other Ph.D. students), non-peers (postdocs and technicians) and supervisors were perceived by students to constitute their primary learning relationships. Each of the three major learning relationships identified in this study were perceived to make an important contribution to doctoral learning processes and in doing so contributed to a community of practice. With only a few exceptions, postdoctoral scientists and technical staff were reported as the primary source of technical learning. This observation is supported by a number of other studies which have reported the importance of non-supervisory peers for Ph.D. students’ technical learning. In a study of doctoral student identity in a neurosciences department, Holley identified technical staff as an important source of assistance with animal-based work [47]. Contrasting doctoral work in the social sciences with that of the laboratory sciences, Delamont, Atkinson and Parry state that (p.329) ‘In laboratory sciences the day-to-day supervision of doctoral research is done, *de facto*, by the postdocs. Whatever a code of practice may say, doctoral students seek routine help and inspiration from postdocs’ [48]. What is interesting in our own data set is the extent to which postdoctoral scientists have supplanted students’ official supervisors in the provision of technical learning. Indeed, only 3 out of 32 students in our cohort (**UK03**, **USA05**, **USA06**) reported learning any practical techniques directly from their supervisors. These findings are in keeping with those of Vilkinas, wherein the (p.304) ‘hands on approach’ adopted by the majority of supervisors interviewed did not involve the teaching of laboratory technique [36].

Compelling evidence now exists to suggest the importance of peer relationships to doctoral learning [17], [28], [31]. In the present study, we identified a qualitative difference in the emotional support provided by inter-student relationships (characterised by an ability to relate to each other’s experiences) and that provided by supervisory relationships (a sense of intellectual confidence and security). Our findings with regards to peer relationships are in agreement with those of Gardner who, in a study based on a cohort of 20 chemistry and history students reported some surprise at (p.736) ‘how frequently and regularly the graduate students mentioned peer support. These comments were spread equally across both programs and peer support was mentioned overall much more frequently than the concept of faculty support’ [49]. Similarly, Martinuso and Turkulainen concluded that (p.117) ‘it seems that peer support plays a strong role in supporting progress in both coursework and research’ [17].

In addition to providing a source of technical learning, we suggest that the value in these relationships lies, at least in part, in the ability of students to be able to empathise with each other’s shared experiences in doctoral programs. This conclusion is supported by a published analysis of doctoral scholarly writing groups by Parker, who concluded that (p.50) ‘one of the factors that appeared to contribute to students’ positive views of the peer review process was the sense of empathy that members of the group had for each other’s work’ [26].

The second aim of this study was to improve our understanding of student perceptions of value in their supervisory relationships. The role of the supervisor has long been held to play a critical role in doctoral learning, and consequently has received a significant amount of attention as a research subject [9], [50]–[52]. More recently, the role of the supervisor in doctoral education has come under increasing scrutiny in response to pressures from institutions, governments, funding agencies and an increasingly market-driven higher education market to improve quantitative indices of doctoral program success including time to completion and non-completion rates [34], [35], [53].

In an elegant phenomenographic study into doctoral supervision published by the Academy of Management Learning & Education in 2007, Wright, Murray and Geale state that (p.458) ‘competence in supervision cannot be reduced to lists of attributes, traits or activities; rather, *how* someone supervises is a manifestation of that person’s holistic understanding of *what* supervision is. At the same time, through enacting the *how* of supervision, supervisors create and recreate the *what* of supervision’ [45]. They further argue that (p.459) ‘to change practice, we must first explore how supervisors make sense of their world. Change requires that supervisors gain an awareness, first, of their own understanding, and, second, of alternate understandings to their own’ [*ibid*]. We argue that as their world includes the students they are supervising, supervisors should also take into account the values placed on the doctoral process, as well as supervisory relationship itself, by the student. Within the setting of African-American doctoral education, Felder and colleagues have advanced Bell’s Concept of interest convergence, (p.4) ‘an element of the advising process whereby a student’s interest converges with the interests of his or her faculty advisor and is supported by the organizational culture’, as an important component the doctoral learning process. In keeping with an increasing appreciation for the complexity of the student-supervisory relationship they note that (p.4) ‘how faculty members and students jointly identify with ideas is essential to understanding the evolution of common interests during the doctoral process’ [54].

In the present study, we identified that students found value in the provision of access to research networks, funding, project guidance and emotional support through their relationships with supervisors. As identified in previous work (including our own), the supervisory role also includes socialising students to research culture and philosophy as well as providing higher-order guidance in the execution of research programs [31], [55]–[57].

Output orientated functions (i.e. obtaining funding and directing project management) of supervision are well established as being important for successful doctoral learning. The identification of their importance to students in the present study is in keeping with earlier studies of doctoral supervision, a comprehensive summary of which is provided by a landmark examination of supervision in a research context Pearson and Brew [29]. More recently, process-orientated supervisory functions, such those described in Wright, Murray and Geale’s (p.207) ‘Quality Assurer’ conception of supervisory practice and are also represented in what McCallin and Nayar refer to as (p.66) ‘the traditional view of supervision that has focused much more on methodological issues’ [34], [45].

The importance of supervisory relationships as a source of emotional support is also well established in the literature. Deuchar refers to a (p.490) ‘pastoral style’ of supervision that takes into account the need for both personal and project-specific support [53]. Concepts of (p.466) ‘trust’, ‘support’, ‘encouraging’, and ‘counselling’ are reflected in Wright, Murray and Geale‘s ‘Supportive Guide’ supervisor [45]. Pearson and Brew note that (p.141) ‘the willingness, and indeed the ability, of supervisors to support and encourage, even when they are worried that a student may be going down successive blind alleys, is a rare skill’ [29].

Although there is a great appreciation for the importance of an emotional element to supervisory practice, limited data exist to describe its form and function. In the present study we contend that the form of emotional support students perceived as being provided by their supervisor(s) was unique and distinct from that identified to exist in relationships established with other members of their learning environment. The hallmarks of this unique emotional support were the provision of a sense of intellectual and academic security.

The doctoral learning environment is, relative to undergraduate or taught-postgraduate courses, highly unstructured. In a further significant departure from previous educational experiences, doctoral students act both as producers of knowledge and arbiters of ways of understanding. In our cohort, students often reported perceiving themselves to be novices in undertaking such activities. In the present study, they reported looking to supervisors, more seasoned researchers, to provide reassurance that their efforts were intellectually and scientifically valid. Interestingly, as well as benefitting from direct positive feedback and encouragement, students appeared to gain some semblance of this support simply by virtue of having an accomplished supervisor who had not adversely commented on their work; as reported by student **UK07:** *So he’s kind of overseeing it and the advantage is that he’s been in it for over forty years so if he was really concerned he’d tell me straight away. So clearly he is not concerned too much.*

Socialising doctoral students to research cultures and practices is a process that has been shown to be key to student learning [25], [49], [58]. This is an element of the doctoral learning environment in which supervisors are believed to play a critical role. Girves and Wemmerus note that (p.171) ‘Faculty are the gate keepers to the scholarly professions. Faculty members are the socializing agents of the discipline; they impart the norms and expectations’ [25]. A lack of trust and intellectual support were two supervisory elements identified by Golde as causing (p.686) ‘much of the attrition in science departments’ [9]. In light of the data presented in this study, we suggest that the provision of a sense of intellectual confidence and security plays an important role in the contribution of supervisory relationships to doctoral student socialisation. It is tempting to speculate that this support may be provided by regular meetings between students and supervisors; indeed, regular meetings have been identified as important in previous studies and were identified as a commonplace event in the student-supervisor relationships in the present study [24]. However, as was the case with student **UK07**, a sense of intellectual security was provided by a supervisor with whom there was very little regular interaction. Although there is undoubtedly benefit to this perception of benign supervisory oversight in some cases, it also comes at a substantial risk in the absence of a regular review of student progress. Or, as noted by Johnson, Lee and Green, (p.136) ‘the supervision relationship is often fraught and unsatisfactory – as much marked by neglect, abandonment and indifference as it is by careful instruction or the positive and proactive exercise of pastoral power’ [59].

As such, we suggest that the best means of providing the student with this form of emotional support is likely to depend on the needs of individual student. Brailsford has suggested that (p.15) ‘universities consider offering workshops for would-be candidates before enrolment so that initial motives for doctoral study can be explored and reflected upon before a candidate embarks’ [60]. We suggest that a similar reflective process may be of use in order to establish the structure of supervision best suited to each candidate’s needs. Similarly, we suggest that such workshops could be provided at the undergraduate level as part of the undergraduate program’s mission of career orientation.

## Conclusions and Implications for Doctoral Learning

In drawing conclusions from this semi-structured interview study of Ph.D. students’ perceptions of learning relationships in the biomedical sciences we must first take into account the limitations of the study design and cohort. Firstly, and perhaps most importantly, the data contained in this report represent a snap shot of student perceptions at one point in time in their doctoral candidacy. There are good data to suggest that many of the elements that comprise each student’s particular learning environment changes (e.g. the function role of the supervisor) across the development of their candidacy [27]. As such, a learning relationship that is perceived important early in a student’s doctoral life (e.g. learning a series of techniques from a postdoctoral researcher) may become markedly less important or indeed change in nature entirely as the student progresses through their candidacy, gaining in confidence and ability. In addition to this, it is important to keep in mind that our data represent a range of reported student value perceptions. Although we have often included the number of students reporting such views in order to assist in their interpretation, it does not mean that every student found a particular reported learning relationship to be of value, or valuable in the same way. Indeed, the need for supervisors to adopt a flexible approach to take into account student-specific differences in supervisory and learning requirements is a well-established area of scholarship in doctoral learning [28]. Lastly, it is important to consider that this study draws on students from two different countries (the UK and the US), working in quite different doctoral program structures, albeit both at elite, research-intensive institutions. This approach was deliberately chosen to allow the authors to assess the broad generalisability of the learning relationships identified in our original, Australian Ph.D. cohort. Whilst acknowledging these differences, we also argue that the core, laboratory-based nature of doctoral programs in both cohorts allows for a meaningful, joint analysis of data to be performed.

A key conclusion that may be drawn from the present study is the need to design biomedical doctoral programs such that they take into account the wide range of learning relationships that are key to student learning. The supervisor is traditionally held to play the key role in doctoral learning. In light of the findings in the present study we suggest, as indeed have a number of other investigators, that a successful doctorate is predicated upon the student having access to a number of environmental factors that meet their particular learning needs; or framed differently, that effective supervision is just one, albeit high profile, element of a well-functioning doctoral learning environment.

How we, as doctoral supervisors, program coordinators and educators design and implement such a learning environment is a difficult question, especially in a higher education system grappling with increased student numbers, a lack of stable, tenured positions for university staff and reduced government funding [7], [35]. Although not a core part of our learning relationship analysis, a number of students in the US cohort made positive references to the benefits they derived from working in a structured doctoral program. Students commented on the benefits of going through their doctoral studies with a class of fellow Ph.D. students (access to a number of empathetic relationships), having access to a dissertation advisory committee distinct from their PIs or immediate supervisors (project management) and the significant amount of intellectual confidence that they gained by passing the qualifying examination and an oral defence, early in their doctoral candidacy.

For countries such as Australia that operate mostly unstructured doctoral programs, we suggest that further research into how elements of more structured, US and UK-style programs might better support doctoral learning in the biomedical sciences are warranted.
